# Correlates of alcohol use among internally displaced adults in Northern Cross River, Nigeria: a cross-sectional study

**DOI:** 10.4314/ahs.v26i1.9

**Published:** 2026-03

**Authors:** Ugbe Maurice-Joel Ugbe, Peter Bassey Enyievi, Ekpereonne Babatunde Esu, Bernadine Nsa Ekpenyong, Aniekanabasi Jonathan Okon, Emmanuel Onen Ebri

**Affiliations:** Department of Public Health, University of Calabar Nigeria

**Keywords:** Alcohol use, dependence, misuse, internally displaced, conflict, disaster

## Abstract

**Background:**

Forced displacement is high globally as populations are increasingly displaced by conflicts and disasters. Substance misuse is increasingly recognized as a major cause of morbidity and mortality in displaced populations.

**Objectives:**

This study aimed to investigate the correlates of alcohol misuse among internally displaced adults in Northern Cross River, Nigeria.

**Methods:**

A cross-sectional study was conducted in Ogoja displacement settlements. Three-hundred and thirty-five displaced adults were selected using snowball sampling method. Alcohol dependence was measured using the interviewer-administered Alcohol Use Disorder Identification Test (AUDIT) questionnaire. Data were analyzed using descriptive statistics, Chi-square, and multivariable logistic regression.

**Results:**

The prevalence of alcohol misuse was 26.3%. The multivariate analysis revealed that male gender (AOR=7.28; p<0.001), farming occupation (AOR=4.44; p=0.003), discrimination (AOR=3.49; p=0.001), financial strains (AOR=0.12; p=0.002), overcrowded shelters (AOR=3.49; p=0.001), fear of reprisals (AOR=2.49; p=0.016), and concerns about health and safety (AOR=0.087; p<0.001) were significantly associated with alcohol dependence among IDPs in this study.

**Conclusion:**

These findings indicate that nearly three out of every ten displaced adults were alcohol-dependent as a result of traumatic events, life events, and deprivation of basic care. Protection, social, and psychological remedies are required to foster relief and reintegration efforts for internally displaced persons in Nigeria.

## Introduction

Over 33 million persons are displaced by wars and disasters globally^1^. Further perusal into new global displacements indicates that 8.5 million persons and 24.9 million persons were recently displaced by conflicts/violence and disasters, respectively. Over 24% of recent displacements have occurred in Sub-Saharan Africa owing to myriads of war and conflict events as well as natural disasters in the region^1^. In Africa, there is a higher concentration of displacement as a result of unfortunate political crises, wars, religious crises, and communal clashes. Nigeria ranks as one of the top 10 global sufferers of displacement as over 2 million persons have been forced to leave the confines of their homes to seek abode in neighboring communities and villages where they face secondary displacement^2^. Despite the government's efforts, Boko Haram terrorists and other sects in the North-Eastern and North-Western parts of Nigeria have intensified attacks that have led to massive displacement of individuals from their ancestral homes. Forcibly displaced persons can be exposed to a significant number of traumatic events which may cause them to adopt coping strategies such as alcohol use and misuse. A researcher^3^ found that an estimated 20-30% of the Nigerian population suffer from mental disorders and a higher proportion of these disorders exist among the displaced persons across the nation. Some authors^4^ found a high prevalence of depression and anxiety in similar settings. Although infrequently reported, substance use was a coping strategy associated with displacement and was also identified as a comorbidity of Post-traumatic disorder (PTSD) as well as other mental health problems^5^. Alcohol is a frequently used substance that is perceived to remedy painful memories^6^. Loss of social standings, discrimination, legal protection, shelter, economic standing, and issues with re-establishing livelihood are major drivers of alcohol abuse among IDPs^7^. The probability that IDPs could resort to alcohol use as a way out of misery has posed a serious concern to the Nigerian government over the years^8^. This gets worse when children grow up in these environments while following in the steps substance abusers with the insurgency-related conflicts and military operations significantly increasing the prevalence of the substance use^9^.

Substance utilization among IDPs in various parts of Nigeria isa neglected area of research. The IDPs who lose their social, economic, and legal privileges usually suffer considerable physical and psychosocial hardship. They are exposed to special difficulties not experienced by other areas^7^. The World Food Programme stated that specifically, these difficulties are related to re-establishing livelihoods in camp or camp-like settings when traditional means of livelihood are no longer viable. Secondly, with their special needs still unattended, there is a growing consensus that IDPs may not be recommended for special treatment^7^. This loss of livelihoods and social and legal standings have been presumed to be factors leading to alcohol and other substance use among this population.

A systematic review on substance use among conflict-affected populations^10^ found substance misuse and related harm: consumption of excessive alcohol among IDPs in Urban Colombia; intake of alcohol among skilled Sudanese refugees in Kenya; inbreathing of opioids and heroin among asylum seekers in the Middle East; and oral benzodiazepine use in Bosnia and Herzegovina. A study^10^ also found that there was an increased prevalence of HIV risk behaviours among alcohol and injecting substance users in displaced settings. Also, displaced persons and other key informants in relevant studies asserted that high alcohol consumption by all genders led to gender-based violence in East African countries^11^. Furthermore, alcohol and substance use were also associated with heightened risky sexual behaviour which also increased the pathogenesis of sexually transmitted infections, including HIV, around camps and host communities^11^. Substance dependence is highly prevalent among male IDPs than their female counterparts. This may be true because female alcohol and drug users were more hidden, if not less numerous than men. Aside from being male, other factors that may be associated with developing alcohol and drug dependence among displaced persons include exposure to war trauma and the existence of co-existing mental health problems^10^. These factors were also reported in a study^12^ which found that due to frustration, there were increases in smoking and alcohol intake by IDPs in Benue state, Nigeria. However, for these populations, the correlation between humanitarian actions regarding displacement and the promotion of or protection from problem substance use may also have pertinent implications. There is a paucity of research on alcohol use among internally displaced persons in Nigeria. Moreover, Ogoja plays host to many displaced persons whom the government, individuals, and institutions may have failed to recognize. No research of this nature has been undertaken to review the mental capacity of IDPs in terms of alcohol use. It is on this premise that this study was undertaken.

## Methods

### Study design and setting

This descriptive cross-sectional study was conducted among adult (18 years and above) internally displaced persons who reside in settlements in the host communities in Ogoja Local Government Area (LGA), Cross River State, Nigeria^13^. The study was conducted among internally displaced persons (IDPs) residing in settlements located in Northern Cross River, Nigeria. The displaced individuals in these settlements primarily originate from border Local Government Areas (LGAs) and have been affected by various forms of displacement, including communal conflicts, inter-state disputes, and natural disasters. The population includes individuals from LGAs such as Boki, Odukpani, Obubra, Obanliku, and Bakassi, as well as neighboring regions. Sample Size determination

The sample size for this study was determined using the formula for estimating sample size for cross-sectional descriptive studies as cited in Charan and Biswas^14^.


$$n=\frac{Z\alpha 2\;p\;q}{d2}$$

and considering the proportion of internally displaced persons from a previous study as 64%^15^ and 5% precision at 95% confidence interval (CI). Allowance of 10% was made for non-responses and that gave a total sample size of 366.

### Sampling Procedure

Since the respondents did not reside in defined settlements, purposive sampling was used to select three wards majorly inhabited by IDPs. All 10 communities in these 3 wards were selected. We carried out a snowballing sampling to identify the settlements of the hard-to-reach respondents. Only one eligible adult (18 years and above) IDP in each household who consented to participate was recruited. In household were there are more than one adult, simple random sampling technique by balloting was used to settle one eligible adult

### Selection Criteria

Only internally displaced persons who were 18 years and above and settled in communities in the 3 selected wards in Ogoja LGA.

### Instruments for data collection

A semi-structured questionnaire was employed in the collection of Socio-demographic information of respondents and displacement-related factors of Alcohol dependence. Alcohol misuse was measured using the Alcohol Use Disorder Identification Test (AUDIT) questionnaire, a simple and effective 10-item case-finding instrument. The World Health Organization (WHO) created the 10-item Alcohol Use Disorders Identification Test (AUDIT) to evaluate alcohol usage, drinking habits, and alcohol-related issues^16^.

### Method of data collection

Data assistants collected the data from respondents using Open Data Kit (ODK) Collect, a mobile data collection tool that can be linked with an electronic database for data collection and processing. Participants in the study were interviewed directly by trained enumerators and responses were recorded on the Android devices. Data were collected from 92% of the initial calculated sample. Attrition of respondents was as a result of the job engagements and refusal to give consent. However, with a high response rate, this did not affect the bearing of the study's results.

### Method of data analysis

Data were imported into Microsoft Excel and subsequently imported into Statistical Package for the Social Sciences (SPSS) Version 23 after cleaning. No missing data were recorded. The alcohol abuse questions were computed as Liker scale items were respondents were expected to select from 5 options ranging from ‘Never’, ‘monthly or less’, ‘2-4 times a month’, ‘2-3 times a week’ or ‘4 or more times a week’. Alcohol usage that is dangerous or harmful is thought to be indicated by a score of 8 or above. The AUDIT is appropriate for use in primary care settings because it has been validated across genders and in a variety of racial/ethnic groups [16]. A cut-off score of 8 or more indicates hazardous or harmful drinking. The sociodemographic characteristics of respondents, displacement-related factors associated with the outcome and the prevalence of the outcome were analyzed using frequencies and percentages. Pearson's Chi-Square of independence was used to test bivariate associations between the predictors and outcome. Multivariate logistic regression analysis was used to estimate the odds of alcohol dependence with displacement-related factors while controlling for statistically significant covariates. Adjusted Odds Ratios (AORs) were determined with a 95% confidence interval (CI). These factors were selected a priori according to presented evidence from relevant literature and our theoretical presumption. Only factors that were associated with alcohol use disorder in the bivariate procedures were included in the corresponding multivariable procedure while limiting the probable risk of over-adjusting. The significance threshold was set at α = 0.05.

### Ethical Approval

Ethical approval for this study was obtained from the Ethics and Research Committee of the Cross River State Ministry of Health (CRS/MH/HREC/2021/VOL.V1/2010). The study protocols were also in accordance with the 1964 Declaration of Helsinki. Verbal consent was also obtained from the respondents before participation. Participants were also assured of the strict confidentiality of their data. They were also informed that they were not being compelled to be recruited and could opt out at any point in time.

## Results

The prevalence of alcohol dependence in the study population was 26.3%. The demographic characteristics of the respondents are presented in Table 1. Above half of the respondents (51%) were females. The average age of participants was 34.1 (SD=11.1) years. Most respondents (63.3%) were young adults. Most respondents were married (43.3%). Most of the respondents had attained secondary education (36.4%). The majority of the respondents (51.9%) were indigenes of Ogoja. In terms of occupation, the study respondents were predominantly farmers (45.4%). Most of the respondents (60.3%) had less than 4 members living in their households. Slightly below a quarter of respondents (23.3%) earned no monthly income.

**Table 1:** Demographic characteristics of respondents

Variables	Frequency (n=335)	Per cent (%)
**Sex**		
Male	164	49.0
Female	171	
**Age (in years)**		
18-35	212	63.3
36-55	107	31.9
>55	16	4.8
**Marital Status**		
Single	87	26.0
Married	145	43.3
Divorced	22	6.6
Separated	50	14.9
Widowed	31	9.3
**Level of Education**		
No formal education	68	20.3
Primary	83	24.8
Secondary	122	36.4
Tertiary	62	18.5

**Residence Status**		
Indigene	174	51.9
Non-indigene	161	48.1
**Occupation**		
Unemployed	75	22.4
Public Servant	26	7.8
Business Owner	82	24.5
Farmer	152	45.4

**Family size**		
1-3	202	60.3
≥4	133	39.7

**Monthly Income**		
No income earned	78	23.3
Below minimum wage	198	57.9
Within minimum wage	46	13.7
Above minimum wage	9	2.7
Won't say	8	2.4
**Duration of displacement**		
About 1 month	22	6.6
Greater than 1 month to less than 6 months	91	27.2
Greater than 6 months to 12 months	162	48.4
Greater than 12 months	60	17.9

Table 2 indicates the test of association between demographic characteristics and alcohol dependence. There was a statistically significant relationship between Gender and alcohol dependence, *χ*^2^= 41.4, df= 1, p<0.001. Also, there was a statistically significant relationship between age and alcohol use disorder, *χ*^2^ = 7.82, df= 2, p= 0.020. This Chi-square test also identified a statistically significant relationship between the level of education and alcohol use disorder in the study population, *χ*^2^ = 22.1, df= 3, p<0.001.

**Table 2:** Chi-square analysis of the relationship between demographic characteristics and Alcohol dependence

Variables	Presence of Alcohol Dependence (N, %)	Absence of Alcohol dependence (N, %)	p-Value
**Sex**			<0.001**
Male	69 (78.4)	95 (38.5)	
Female	19 (21.6)	152 (61.5)	
**Age (in years)**			0.020*
18-35	52 (59.1)	160 (64.8)	
36-55	27 (30.7)	80 (32.4)	
>55	9 (10.2)	7 (2.8)	
**Marital Status**			0.280
Single	20 (22.7)	67 (27.1)	
Married	46 (52.3)	99 (40.1)	
Divorced	4 (4.5)	18 (7.3)	
Separated	13 (14.8)	37 (15.0)	
Widowed	5 (5.7)	26 (10.5)	
**Level of Education**			<0.001**
No formal education	26 (29.5)	42 (17.0)	
Primary	32 (36.4)	51 (20.6)	
Secondary	23 (26.1)	99 (40.1)	
Tertiary	7 (8)	55 (22.3)	

**Residence Status**			0.291
Indigene	38 (43.2)	123 (49.8)	
Non-indigene	50 (56.8)	124 (50.2)	
**Occupation**			0.030*
Unemployed	15 (17)	60 (24.3)	
Public Servant	2 (2.3)	24 (9.7)	
Business Owner	22 (25)	60 (24.3)	
Farmer	49 (55.7)	103 (41.7)	
**Family size**			<0.001**
1-3	38 (43.2)	164 (66.4)	
≥4	50 (56.8)	83 (33.6)	
**Monthly Income**			0.422
No income earned	19 (21.6)	59 (23.9)	
Below minimum wage	53 (60.2)	141 (57.1)	
Within minimum wage	14 (15.9)	32 (13.0)	
Above minimum wage	0 (0.0)	9 (3.6)	
Won't say	2 (2.4)	6 (2.4)	
**Duration of displacement**			0.283
About 1 month	6 (6.8)	16 (6.5)	
Greater than 1 month to less than 6 months	20 (22.7)	71 (28.7)	
Greater than 6 months to 12 months	50 (56.8)	112 (45.3)	
Greater than 12 months	12 (13.6)	48 (19.4)	

* Statistical significance based on *p*-Value< 0.05

** Statistical significance based on *p*-Value<0.001

There was also a statistically significant relationship between respondents' occupation and alcohol use disorder, *χ*^2^ = 8.96, df= 3, p= 0.030. In terms of family size, there was a statistically significant relationship between the number of persons in the household and alcohol use disorder, *χ*^2^ = 14.6, df=1, p<0.001.

Table 3 indicates the distribution of chi-square p-values to establish the relationship between displacement-related factors and alcohol dependence in the study population. The displacement-related factors significantly associated with alcohol use disorder in the study population include discrimination as a result of present status, *χ*^2^ = 22.4, df=1, p<0.001; financial situation, *χ*^2^= 9.07, df= 1, p=0.003; loss of loved ones due to crises or disaster, *χ*^2^= 12.1, df= 1, p<0.001; living in an overcrowded shelter, *χ*^2^= 39.1, df= 1, p<0.001; fears over reprisal attacks or natural disasters, *χ*^2^= 31.7, df= 1, p<0.001; and concerns about health and safety, *χ*^2^= 17.5, df= 1, p<0.001.

**Table 3:** Chi-square analysis of displacement-related factors associated with alcohol use disorder

Items	Alcohol dependence response categories		
	Yes N (%)	No N (%)	*p*-Value
Work Stress	66 (75.0)	22 (25.0)	0.810
Discrimination	48 (54.5)	40 (45.5)	<0.001**
Financial strain	77 (87.5)	11 (12.5)	0.003*
Lost loved ones in crises or natural disasters	55 (62.5)	33 (37.5)	0.001*
Affected by an outbreak of diseases	81 (92.0)	7 (8.0)	0.063
Living in an overcrowded shelter	65 (73.9)	23 (26.1)	<0.001**
Fears over reprisal attacks	60 (68.2)	28 (31.8)	<0.001**
Separated from family due to conflicts and/or disasters	48 (54.5)	40 (45.5)	0.141
Lack of basic amenities (food, potable water, etc.)	7 (8.0)	81 (92.0)	0.131
Experienced domestic violence	16 (18.2)	72 (81.8)	0.072
Concerned about health and safety	73 (83.0)	15 (17.0)	<0.001**

* Statistical significance based on *p*-Value< 0.05

** Statistical significance based on *p*-Value<0.001

Table 4 shows the multivariate logistic regression analysis of the Sociodemographic correlates and displacement-related factors associated with alcohol use disorder after adjusting for all potential covariates that were found significant in the bivariate chi-square analysis. Males were more likely than females to misuse alcohol in the study population, b = 1.99, p< 0.001, AOR = 7.28 (95% CI: 3.41, 15.6). Age showed no significant relationship with this outcome. Occupation showed a significant relationship with the outcome. More specifically, farmers were about 4.44 times more likely than others to develop alcohol use disorders, b = 1.49, p = 0.003, AOR = 4.44 (95% CI: 1.65, 11.95). Respondents who reported being discriminated against due to their displacement status were about 3.5 times more likely than those who were not discriminated against to be alcohol-dependent, b = 1.26, p = 0.001, AOR = 3.53 (95% CI: 1.66, 7.52). Also, displaced persons with no financial constraints were more likely than those experiencing financial constraints to develop alcohol use disorder, b = -2.15, p< 0.002, AOR = 0.12 (95% CI: 0.03, 0.46).

**Table 4:** Multivariate analysis of correlates of alcohol dependence in the study population

Variables	AOR	95% CI	*P*-Value
**Sex**			
Female	1	-	-
Male	7.28	3.41, 15.6	< 0.001**
**Age (in years)**			
18-35	1	-	-
36-55	0.68	0.32, 1.41	0.291
>55	1.96	0.49, 7.84	0.342
**Occupation**			
Unemployed	1		
Public Servant	1.83	0.29, 11.6	0.522
Business Owner	1.49	0.53, 4.2	0.452
Farmer	4.44	1.65, 11.95	0.003*
**Family size**			
1-3	1	-	-
≥4	0.92	0.45, 1.86	0.813
**Discriminated because of the present status**			
No	1	-	-
Yes	3.53	1.66, 7.52	0.001*
**Financial Strain**			
No	1	-	-
Yes	0.12	0.030, .456	0.002*
**Loss of loved ones**			
No	1	-	-
Yes	1.21	0.59, 2.46	0.604
**Overcrowded shelter**			
No	1	-	-
Yes	3.49	1.72, 7.11	0.001*
**Fears over reprisal attacks or natural disasters**			
No	1	-	-
Yes	2.49	1.18, 5.27	0.020*
**Concerns about health and safety**			
No	1		

Furthermore, this study found that displaced persons who lived in overcrowded shelters were almost 4 times more likely than those who did not live in overcrowded shelters to develop this outcome. However, there was a significant relationship between poor living conditions and the outcome. A significant relationship was identified between fear of reprisal attacks and/or natural disasters and alcohol misuse, b= 0.92, p = 0.02, AOR =2.49 (95% CI: 1.18, 5.27), implying that displaced persons who reported fear were over twice more likely than those who did not report fear of reprisal attacks and reoccurrence of natural disasters to be symptomatic to the outcome. Finally, displaced persons who were concerned about their health and safety were more likely than those who were not concerned to misuse alcohol, b= 1.26, p< 0.001, AOR = 1.87 (95% CI: 1.24, 2.31).

## Discussion

Overall, the results of this present study showed a moderate prevalence of alcohol dependence in the study population. Studies have identified the frequent use of drugs and alcohol among displaced persons globally. This finding and its prevalence figure were consistent with findings by Maigida and Hassan^17^ which identified the prevalence of any substance used to be 25.4% among IDPs in North-Central, Nigeria. Similarly, Usoro, Okediji^18^ in their study found that the prevalence of any substance use was 48.3% among IDPs in Akwa Ibom State, Nigeria. Furthermore, a Georgian study found the prevalence of alcohol use to be 29% among displaced persons^19^. These similarities in prevalence across different geographical lines exist regardless of the instruments used for the respective studies. Generally, alcohol and drug use disorders have shown lower prevalence than other mental problems among IDPs in most studies. This may be due to various country-wide and/or religious/cultural laws which prohibit the use of several substances in the general population; hence the distribution of these substances is limited.

Our study found that males were more likely than their female counterparts to be alcohol-dependent. Being female was however protective of any alcohol use disorder. This may be explained by men's need for social inclusion in their communities. Also, this may be used as a coping strategy to forget about life's stressors^19^. Discrimination was a factor significantly associated with alcohol misuse in the study population. A possible reason could be that prolonged periods of discrimination spark up a desire to not care or think and also leads to frustration. These populations however tend to recognize alcohol use as a coping strategy. This is consistent with a few studies^18^, ^20^. Displaced persons may also indulge in alcohol or other substance use as coping mechanisms to deal with the poor living conditions and fears of reprisal attacks or reoccurring disasters. This is consistent with similar studies that carried out research in IDP camps^21^-^23^.

Interestingly, those who experienced financial pitfalls were at a lower risk of indulging in alcohol misuse. In essence, displaced persons who had no financial strains engaged more and were identified to have symptoms of alcohol dependence. To the best of our knowledge, no previous studies have contrasted or supported this finding. Lastly, those who were not concerned about their health and safety were more likely than those concerned to be symptomatic of alcohol use disorder. This may be a result of displaced persons' perceptions of caring less about life and indulging in risky behaviours since they also perceive that they have nothing to lose. No previous studies have been found to support or refute this finding.

## Implications

Findings from this study show that several factors combine to significantly predict excessive use of alcohol among internally displaced persons. Though financial strain was found to be protective, it does not reflect a good socioeconomic standing for respondents but rather contributes to a lower intake of harmful alcoholic products. However, mental health intervention studies are necessary to better understand this association in future research.

## Limitations

This is a cross-sectional study; hence, data should be read with caution. Data were self-reported, thus creating significant bias in data collection. There is also a risk of responder bias since some respondents may perceive the research to be a poverty alleviation programme, thus drawing bias responses.

## Conclusion

This research reveals a pressing public health concern, with 26.3% of internally displaced adults in Ogoja, Nigeria, exhibiting alcohol dependence, fueled by displacement-related stressors. Male gender, farming occupation, discrimination, financial difficulties, overcrowded shelters, fear of reprisal attacks, and health and safety anxieties emerged as significant drivers of alcohol misuse. These findings highlight the critical need for comprehensive interventions—integrating mental health support, economic empowerment, and improved living conditions—to address the root causes of substance dependence. Coordinated efforts across government, community, and humanitarian sectors are vital to restore dignity, enhance resilience, and support sustainable reintegration for these marginalized populations
